# Reversing the Intractable Nature of Pancreatic Cancer by Selectively Targeting ALDH-High, Therapy-Resistant Cancer Cells

**DOI:** 10.1371/journal.pone.0078130

**Published:** 2013-10-23

**Authors:** Sang Kyum Kim, Honsoul Kim, Da-hye Lee, Tae-shin Kim, Tackhoon Kim, Chaeuk Chung, Gou Young Koh, Hoguen Kim, Dae-Sik Lim

**Affiliations:** 1 Graduate School of Medical Science and Engineering, South Korea Advanced Institute of Science and Technology (KAIST), Daejeon, South Korea; 2 Department of Radiology, Yonsei University, College of Medicine, Seoul, South Korea; 3 Department of Biological Sciences, KAIST, Daejeon, South Korea; 4 Department of Pathology, Yonsei University, College of Medicine, Seoul, South Korea; Center for Molecular Biotechnology, Italy

## Abstract

Human pancreatic ductal adenocarcinoma (PDAC) is a cancer with a dismal prognosis. The efficacy of PDAC anticancer therapies is often short-lived; however, there is little information on how this disease entity so frequently gains resistance to treatment. We adopted the concept of cancer stem cells (CSCs) to explain the mechanism of resistance and evaluated the efficacy of a candidate anticancer drug to target these therapy-resistant CSCs. We identified a subpopulation of cells in PDAC with CSC features that were enriched for aldehyde dehydrogenase (ALDH), a marker expressed in certain stem/progenitor cells. These cells were also highly resistant to, and were further enriched by, treatment with gemcitabine. Similarly, surgical specimens from PDAC patients showed that those who had undergone preoperative chemo-radiation therapy more frequently displayed cancers with ALDH strongly positive subpopulations compared with untreated patients. Importantly, these ALDH-high cancer cells were sensitive to disulfiram, an ALDH inhibitor, when tested *in vitro*. Furthermore, *in vivo* xenograft studies showed that the effect of disulfiram was additive to that of low-dose gemcitabine when applied in combination. In conclusion, human PDAC-derived cells that express high levels of ALDH show CSC features and have a key role in the development of resistance to anticancer therapies. Disulfiram can be used to suppress this therapy-resistant subpopulation.

## Introduction

The prognosis of patients with pancreatic ductal adenocarcinoma (PDAC) is extremely poor. Only 10-20% of PDACs are suitable for surgical resection at initial diagnosis, and the tumor recurrence rate has been reported to reach up to 70-90%, even in patients who have undergone curative resection [1]. The therapeutic options in a majority of patients eventually become reduced to intensified chemotherapy and/or radiation therapy [1,2]. Therefore, the treatment of PDAC is extremely challenging because it easily gains resistance to chemo-radiation therapy and becomes intractable. 

A growing body of research supports the concept of cancer stem cells (CSCs) or tumor-initiating cells. CSCs characterize unique features, including the capacity for unlimited self-renewal, long lifespan, high metastatic potential, and resistance to chemotherapy [3,4]. Thus, CSCs have come to be recognized as a tumor sub-population that should be vigorously targeted [2,3,5]. But as a practical matter, the efficacies of currently available CSC-targeting therapies are far from satisfactory. Several studies have validated the role of CSCs in the development of therapeutic resistance in PDACs [4], and demonstrated that they are often resistant to the most commonly used anticancer drugs, such as gemcitabine and 5-fluorouracil [1,4,6].

In this study, we presumed that the concept of CSCs could explain why the effects of standard chemotherapies are frequently limited in patients who receive adjuvant chemotherapy or chemo-radiation therapy [7-11]. We further hypothesized that certain unique features of CSCs could be exploited to re-sensitize PDACs to anticancer treatments and subsequently reverse the intractable nature; our attention was drawn to aldehyde dehydrogenase (ALDH), which has been recognized as a new CSCs marker especially as part of a panel of stem cell markers [12-18]. Accumulating evidence supports the idea that increased expression of ALDH is associated with adverse prognosis in breast, lung, and prostate cancers [15,16,18]. Adding to this list, Rasheed et al. recently reported that ALDH1-positive pancreatic cancer cells negatively influence the survival of PDAC patients [12].

Disulfiram (1-[diethylthiocarbamoyldisulfanyl]-N,N-diethyl-methanethioamide) is an irreversible inhibitor of ALDH that has been used to achieve alcohol-avoidance behavior during treatment of alcoholics [19]. Disulfiram has gained attention as a candidate anticancer medication and to date has been tested for several solid tumors, such as breast cancer, melanoma, and colorectal cancer [20-22]. Although the precise mechanism underlying the anticancer activity of this agent has not been fully elucidated, several significant clues have emerged [20-26]. Recently, Dalla Pozza et al. reported that the combined treatment with gemcitabine and disulfiram with zinc ion inhibited the xenograft tumor growth of pancreatic cancer cells [27]. However, the direct effect of disulfiram on CSCs of PDACs in relation to ALDH is undetermined. 

In this study, we explored the relevance of ALDH expression and CSC populations in PDAC. We compared responses to disulfiram between tumor sub-populations with different levels of ALDH expression and found that ALDH strongly positive cells were sensitive to disulfiram. Our results suggest the applicability of disulfiram as an anti-CSC agent that could possibly improve the efficacy of standard chemotherapies against PDAC.

## Materials and Methods

### Cell lines and reagents

The following human cell lines were used: CFPAC-1, MIA PaCa-2, PANC-1, and AsPc-1 (pancreatic cancer cell lines); hTERT-HPNE (normal pancreatic ductal epithelial cell line). All the cell lines were purchased from the American Type Culture Collection (ATCC, Manassas, VA).The cell lines have been tested using AmplFLSTR identifier PCR Amplification kit (Applied Biosystems, Foster, CA, cat. 4322288) and authenticated by KCLB in February 2013. Disulfiram and gemcitabine (Sigma) were used for *in vitro* viability and *in vivo* xenograft assays. 

### Human tumor samples

This retrospective study was approved by the institutional review board of Yonsei University Medical Center. Written informed consent was obtained from each patient for storage and use of specimen and medical information. Our inclusion criteria defined a study population of 57 PDAC patients who had been histologically diagnosed and operated Yonsei University Medical Center from 2005 to 2008. Clinical assessment was performed based on American Joint Committee on Cancer TNM (tumor-node-metastasis) classifications. Because stage III and IV pancreatic cancers are unresectable according to TNM classifications [28], we analyzed only stage II PDAC patients who received Whipple’s operation or pylorus-preserving pancreatoduodenectomy with lymph node dissection. For preoperative chemotherapy/CCRT, gemcitabine was used in the majority (18/20; 90%) of patients.

### Flow cytometry

All cell lines were analyzed for the expression of ALDH by flow cytometry using an Aldefluor Kit (Aldagen, Inc.) following the manufacturer’s instructions. For comparison of ALDH-bright and ALDH-negative cells, CFPAC-1 cancer cells were sorted based on the intensity of their ALDH activity measured by FACS (fluorescence-activated cell sorting, BD FACS Aria™ II cell sorter). Biotin-conjugated anti-CD24 and PerCP-Cy5.5 anti-CD44 antibodies (eBioscience) were used according to an immunophenotyping protocol included in the manual provided by the vendor. DAPI (4', 6-diamidino-2-phenylindole) was used to stain the nuclei of live cells. A PE Annexin V Apoptosis Detection kit I (BD Bioscience) was used in conjugation with 7-AAD (eBioscience) to identify early apoptotic cancer cells. For the analysis of the LSK (lineage- Sca+ c-kit+) hematopoietic stem cell frequency in the bone marrow from murine femur and tibia, we used lineage (CD11b-biotin, Gr1-biotin, CD4-biotin, CD8-biotin, B220-biotin, Ter119-biotin)-streptavidin-PerCP-Cy5.5, PEcy7-conjugated sca-1, and APC-conjugated c-kit antibodies (eBioscience).

### Western blotting

Protein extracts were obtained from cells or tumor masses using a protein extract solution (Pro-Prep; iNtRON) following the manufacturer’s instructions. Antibodies used for Western blot analysis included anti-ALDH1A1 (Abcam), GAPDH and anti-β-actin (Sigma).

### Real-time polymerase chain reaction (PCR)

Isolation of RNA and cDNA synthesis were performed following the manufacturer’s protocol (RiboEx; iNtRON). SYBR green-based array PCR was performed using the iQ5 Multicolor Real-Time PCR Detection System (Bio-Rad). The sequences of the various PCR primers are available on request. Relative mRNA expression levels were calculated using the comparative Ct method with normalization to β-actin.

### Mouse experiments

Animal care and experimental procedures were performed with the approval of the KAIST Animal Care and Use Committee. We used 6-week-old male *Foxn1* ^*nu*^ SCID mice for xenograft assays (Orient Bio). Disulfiram tablets (Ind-Swift) were dissolved in corn oil (Sigma) for oral administration via a sonde. Gemcitabine (Sigma) was intraperitoneally injected (I.P) for *in vivo* regression assays. 

For measurement of disulfiram response, we divided the xenotransplanted mice into disulfiram-treated mice (300 mg/m^2^, twice-weekly, administrated via a sonde) and corn oil-treated control mice. For measurement of the efficacy of combination therapy with disulfiram and low-dose gemcitabine, we divided the xenotransplanted mice into five groups as, disulfiram-only treated mice (300 mg/m^2^, twice-weekly, administrated via a sonde), low-dose gemcitabine treated mice (40 mg/m^2^ body surface area, I.P, weekly) [29], low-dose gemcitabine along with oral disulfiram, 10-times higher dose of gemcitabine treated mice (400 mg/m^2^ body surface area, weekly), and PBS-treated control mice (I.P).

It is recommended that oral drug disulfiram is taken an initial dose of 500 mg followed by a daily maintenance dose of 250 mg in human [30]. The dose translation from one animal species to another using the body surface area (BSA) normalization method is recommended [31]. According this formula, we administered disulfiram of 300mg/m^2^ to mice twice a week *per oral*.

### Tumor volume measurements

Mice were enrolled for drug treatment when their tumors had reached a size of at least 200 mm^3^. Tumor size was measured with a caliper, and tumor volume was calculated using the standard formula, length x width^2^ x 0.5 (mm^3^). Tumor initiation in cancer cell-injected mice was monitored daily, and tumor size was determined weekly for 3 to 5 weeks, as indicated.

### Immunohistochemistry

Tumor xenografts were fixed in formalin, embedded in paraffin, sectioned, and stained with hematoxylin and eosin (H&E). Immunohistochemistry (IHC) with the anti-ALDH1A1 antibody (Abcam) was performed using a Dako Envision Kit following the manufacturers’ instructions. We used internal standards to define ALDH1A1 strongly positive cells when the amplitude of signal was equal to or greater than that of normal stem/progenitor cells located in the tumor-free region of the same slide (Figure S1). 

**Figure 1:**
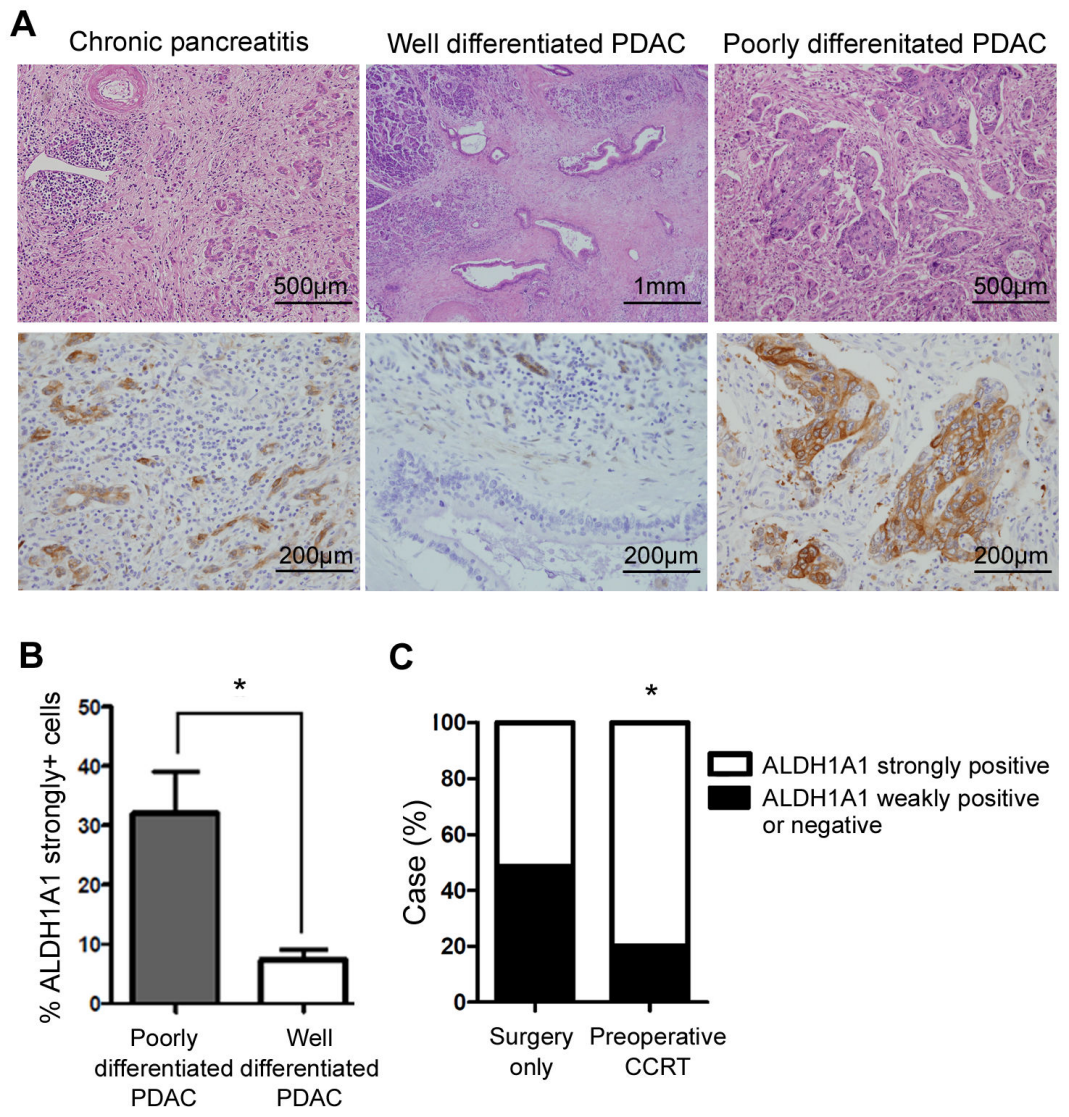
IHC analysis of ALDH1A1 in surgical specimens from human PDAC. (A) IHC analysis of ALDH1A1 in surgical specimens of human chronic pancreatitis and PDAC. Upper panels, H&E stain and lower panels, ALDH1A1 IHC stain. (B) The frequency of ALDH1A1 strongly positive cells between well differentiated and poorly differentiated PDACs. (C) Profiles of ALDH activity of human PDACs according to whether preoperative treatment was performed. *P < 0.05. Error bars represent standard deviations.

We performed IHC analysis of ALDH1A1 and compared glandular differentiation with ALDH1A1 expression on the human PDAC specimen (Figure 1A). To express the correlation numerically, we counted ALDH1A1 strongly positive cancer cells per total cancer cells composed of well differentiated and poorly differentiated glands, respectively. We examined three well differentiated and three poorly differentiated PDAC cases of human in 10 high-power fields (X400 magnification).

### Limiting-dilution analysis

To estimate the frequency of colony-formation, we diluted CFPAC-1 cells into a single cell per well (100µL) and cultured on 96 well plate in 5% CO_2_ incubator at 37°C. After two weeks, we counted each colony outgrowth from a single cell and calculated number of colonies out of total wells. 

### Viability assay

We performed viability assay by WST (water-soluble tetrazolium salt) dye (EZ-cytox cell viability assay kit, itsBIO), according to the vendor’s instruction. Samples were read by a microplate reader using 450nm wavelength of absorbance. To quantify the cell viability, we determined the average values from triplicate or quadruplicate readings and have got standard curve using GraphPad Prism 5 software. 

### Statistics

All statistical analyses were performed using GraphPad Prism 5 software. For the analysis of cell viability and tumor volume according to drug treatment, significant differences between means were determined by an analysis of variance (ANOVA) followed by t-tests. IHC and flow cytometry results were analyzed by t-test and the ratio of tumors containing ALDH-strongly positive cancer cells in surgically-resected PDACs was compared by chi-square method. We compared colony forming efficiency by an analysis of t-test. All reported P-values are two-sided, and P-values less than 0.05 were considered to indicate statistical significance.

## Results

### ALDH1 expression in a subtraction of tumorous and non-tumorous human pancreatic tissues

Terminal ductal cells have been reported as pancreatic stem/progenitor cells in several studies [32-34] and Rovira et al. characterized the ALDH1 expression pattern of these cells in mice [35]. However, the expression pattern of ALDH1 in non-tumorous human pancreatic tissue has not been reported. We thus examined human specimens and found that, similar to the murine pancreas, human chronic pancreatitis exhibited regenerative metaplasia and proliferating ductal cells which would be the immediate progeny of the pancreatic stem/progenitor cells [32,35] {Rovira, 2010 #96}also showed high levels of ALDH1A1 expression (Figure 1A). 

In human PDAC, ALDH1A1 expression varied among different cells across a wide spectrum from negative to strongly positive (Figure S1). Notably, poorly differentiated PDACs displayed more abundant ALDH1A1 strongly positive cancer cells than well-differentiated PDACs did (Figure 1A). Figure 1B indicates the statistical difference in frequency of ALDH1A1 strongly positive cells between well differentiated (n=7) and poorly differentiated (n=7) PDACs. We counted the cancer cells in the 10 high-power fields (X400) of the representative slide from each of PDAC specimens.

Next, we examined the surgical specimens who either received preoperative chemotherapy/concurrent chemo-radiation therapy (CCRT; n = 20) or not (n = 37), and searched for the presence of ALDH1A1 strongly positive (Table S1). These cells were identified by comparing the ALDH1A1expression level of an individual cancer cell to that of a normal stem/progenitor cell included in the adjacent tumor-associated chronic pancreatitis tissue (Figure S1). We found that the frequency of tumors that possess ALDH strongly positive cells were higher in the patients who received chemotherapy/CCRT prior to surgery (80.0%) when compared with that of the patients who received surgical resection alone (51.4%) (Figure 1C), suggesting that ALDH strongly positive cancer cells were enriched by conventional chemotherapy/CCRT.

### Cancer cells that highly expressed ALDH display multiple CSC features

To establish an *in vitro* platform, we screened one normal human pancreatic ductal epithelial cell line (hTERT-HPNE) and four human PDAC-derived cell lines (CFPAC-1, MIA PaCa-2, PANC-1 and AsPc-1). We measured ALDH activity in each cell line by flow cytometry using Aldefluor kit (Figure S2). Because ALDH1A1 is a major product of the *ALDH* gene, fluorescence intensity should positively correlate with ALDH1A1 [36]. Thus, a subpopulation of cells highly expressing ALDH in flow cytometry could be correspond with the ALDH1A1 strongly positive cells observed in human PDAC specimens.

We defined three subset of cells based on their flow cytometric distribution relative to the gating of DEAB negative control either as ALDH-negative (cells located inside the region gated by DEAB control), ALDH-high (cells located in the region over DEAB control), and ALDH-bright (a subpopulation of ALDH-high cells). In hTERT-HPNE, the ALDH highly expressing subset constituted less than 5% of the total population. Among the four PDAC cell lines, MIA PaCa-2 (>90% of the total population) and CFPAC-1 (approximately 50%) demonstrated abundant ALDH-high cancer cells. In PANC-1 and AsPc-1 cells, the ratios of ALDH-high populations were comparable to or less than that of hTERT-HPNE cells. Consistently, ALDH1A1 protein was detected in significant amounts in MIA PaCa-2 and CFPAC-1 cells lines, but only at minimal levels in PANC-1 and AsPc-1 cells (Figure S2). 

On the basis of previous literature [12-14,18,37] and our observations, we inferred that ALDH-bright cancer cells might actually represent CSCs. Previous studies have applied ALDH, as part of a panel with other stem cell markers, to identify normal and cancer stem cells in the pancreas [12,13,37]. We tested whether ALDH alone can be used as a bona fide stem cell marker and whether cells with high levels of ALDH expression actually represented CSCs. To do this, we used CFPAC-1 cell line because it is known that the subpopulation of CFPAC-1 is enriched with cancer stem-like cells [38] and we found that CFPAC-1 showed evenly distributed ALDH-high and -negative cancer cells in Aldefluor assay. We further sorted cells based on the magnitude of their ALDH activity identified by FACS and compared the upper and lower 5% of cells, which we defined as ALDH-bright and ALDH-negative sub-populations, respectively. ALDH1A1 mRNA and protein levels were consistent with ALDH levels identified by FACS (Figure S2).

**Figure 2:**
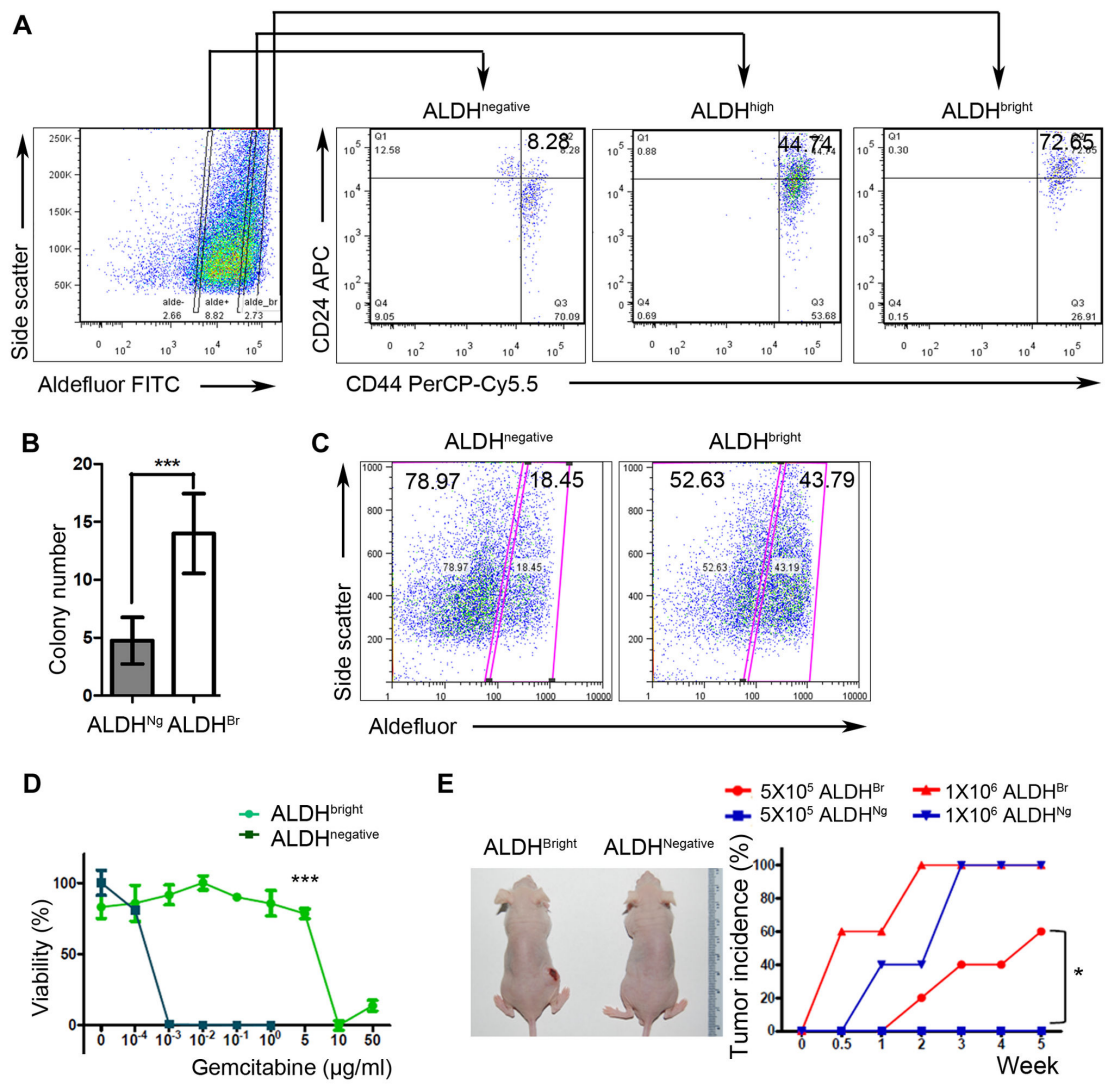
ALDH strongly positive CFPAC-1 cells represent a subpopulation that shows CSC features. (A) FACS analysis for CD24^+^CD44^+^ CFPAC-1 cells categorized into ALDH^bright^, ALDH^high^ and ALDH^negative^ groups. (B) Limiting dilution colony-forming assays performed with FACS-sorted CFPAC‑1 cells. (C) ALDH activity measured by FACS analysis in ALDH^negative^ and ALDH^bright^ CFPAC-1 cells cultured separately for 2 weeks. (D) Assay of ALDH^bright^ and ALDH^negative^ CFPAC-1 cell viability after treatment with gemcitabine. (E) Representative image and graph comparing tumor incidence after injection of FACS-sorted ALDH^bright^ or ALDH^negative^ CFPAC-1 cells. Mice were injected with the indicated number of cells (ALDH^Br^, 5 x 10^5^[*n* = 5]; ALDH^Ng^, 5 x 10^5^[*n* = 5]; and ALDH^Ng^, 1 x 10^6^ [*n* = 5]; ALDH^Br^, 1 x 10^6^ [*n* = 10]) on the right flank and observed for 5 weeks. 1X10^5^ALDH^negative^ cancer cells failed to generate any nodule, even when the observation period was extended up to 14 weeks. ALDH^Br^, ALDH-bright and ALDH^Ng^, ALDH-negative. *P < 0.05 and ***P < 0.0005.

We next analyzed CFPAC-1 cells with different levels of ALDH for the co-expression of CD24 and CD44, which are commonly used stem cell markers [14]. This revealed that ALDH activity positively correlated with those of CD24 and CD44 (Figure 2A). A limiting dilution colony-forming assay revealed that ALDH-bright cells had greater colony-forming activity than ALDH-negative cells (Figure 2B). We separately cultured ALDH-bright and ALDH-negative cells, and analyzed the ALDH activity after 2 weeks (Figure 2C). The progeny of ALDH-bright cells included both ALDH-bright and ALDH-negative cells; thus, ALDH-bright cells successfully re-established the original population. In contrast, ALDH-negative cells failed to fully recover the ALDH-bright cell population. 

Another well-characterized feature of CSCs is their resistance to chemotherapy and/or radiation-therapy [3]. Hence, we assessed the sensitivity of ALDH-bright and -negative cancer cells to a conventional anticancer drug, gemcitabine. ALDH-bright cancer cells were highly resistant to gemcitabine compared with ALDH-negative cancer cells, requiring significantly higher concentrations to kill 50% of cells (the half maximal inhibitory concentration, IC_50_) (Figure 2D). This result is compatible with those of human data that ALDH strongly positive cancer cells were enriched by conventional chemotherapy/CCRT (Figure 1C).

Next, we subcutaneously injected 5 x 10^5^ FACS sorted CFPAC-1 ADLH-bright and -negative cancer cells into the flanks of nude mice, respectively. The ALDH-bright xenografted cells began to form a nodule within 1 week after injection, and by 5 weeks 60% of mice had successfully formed a nodule (Figure 2E). However, the same number of ALDH-negative cancer cells failed to generate any nodule, even when the observation period was extended up to 14 weeks. ALDH-negative cancers were able to establish tumor nodules only when the injection number of cells was increased to 1 x 10^6^. On histologic examination, the xenograft nodules originated from ALDH-bright cancer cells exhibited structures with various degrees of differentiation, ranging from well differentiated cells that successfully formed organized glands with a lumen to poorly differentiated cells that barely formed abortive glandular architectures (Figure S2). Tumor cells located within poorly differentiated regions frequently exhibited a relatively atypical and pleomorphic morphology and displayed bizarre nuclei compared with those from well-differentiated areas. IHC for ALDH1A1 showed more strongly positive cells in the solid sheet than in well-formed glands (Figure S2), in a manner consistent with those of human PDAC specimens (Figure 1A-B).

These results indicated that ALDH levels is sufficient to define a subpopulation of pancreatic cancer cells that demonstrates poor differentiation, repopulating abilities, resistance to gemcitabine, and greater tumorigenicity, which are compatible with the typical features of CSCs.

### ALDH-bright PDAC cells are selectively and efficiently eliminated by disulfiram

**Figure 3:**
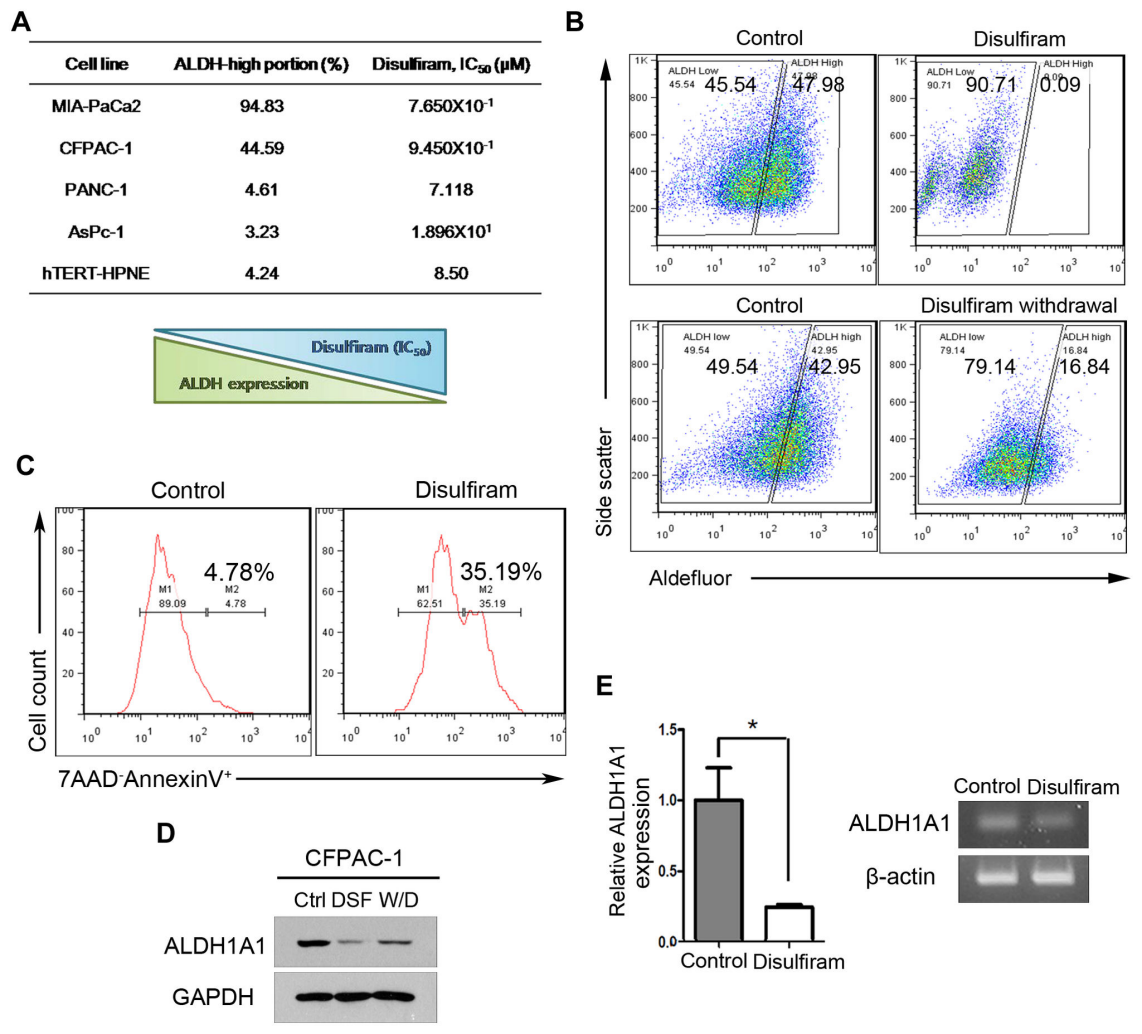
PDAC-derived cell lines with higher ALDH activity are more sensitive to disulfiram. (A) IC_50_ of disulfiram according to ALDH expression. (B) Aldefluor assay of ALDH activity in CFPAC-1 cells treated with disulfiram (top panels) or treated transiently with disulfiram followed by drug withdrawal (bottom panels). (C) Flow cytometric assay for 7AAD^-^AnnexinV^+^ early apoptotic cells after 12-hour treatment with disulfiram (10μM). (D) Representative Western blots showing ALDH1A1 protein levels in CFPAC-1 under control conditions, disulfiram-treated cells, and in transiently disulfiram-treated cells with subsequent withdrawal of disulfiram. (E) RT-PCR and qRT-PCR measurements of ALDH1A1 mRNA expression in control and disulfiram-treated cells. *P < 0.05.

Because we identified ALDH-bright cancer cells as highly gemcitabine-resistant, we suspected that ALDH strongly positive cells were responsible for chemotherapy resistance. To selectively eliminate ALDH-bright cells, we turned to disulfiram, which is a well-known irreversible inhibitor of ALDH [19], and investigated the correlation between the level of ALDH expression and disulfiram sensitivity in all four human PDAC cell lines. We treated disulfiram and calculated IC_50_ values based on viability assays. We observed an inverse correlation between the percentage of cells expressing ALDH and IC_50_ values for disulfiram (Figure 3A and Figure S3). 

The toxicity of chemotherapeutic agents on normal tissue is another major consideration during anticancer treatment. Therefore, we simulated the toxicity of disulfiram by measuring their effects on a normal pancreatic epithelial cell line, hTERT-HPNE (Figure S3). hTERT-HPNE cells were largely insensitive to relatively high concentrations of disulfiram. Notably, the IC_50_ of disulfiram for hTERT-HPNE cells by far exceeded those of Mia PaCa-2 and CFPAC-1 cells, indicating that a sufficiently wide therapeutic window can be secured. In contrast to the effects of disulfiram on MiaPaCa-2 and CFPAC-1 cells, the IC_50_ values of disulfiram for PANC-1 and AsPc-1 cancer cell lines were very high, a difference that we attribute to the low percentage of PANC-1 and AsPc-1 cells that express ALDH. 

Next, we further examined the effects of disulfiram on ALDH-bright cancer cells, by assessing ALDH levels by flow cytometry in the remaining viable CFPAC-1 cancer cells after treatment with disulfiram (10 μM) for 12 hours (Figure 3B). These experiments revealed that disulfiram did not significantly affect the ALDH-low population. To test whether the disulfiram effect was sustained even after it had been removed, it had been withdrawn from the media and the cells were subsequently cultured for additional 2 weeks. Interestingly, after disulfiram had been withdrawn, the cells failed to recover the original tumor hierarchy and exhibited a persistent shift to the left side of the flow cytometric distribution. Moreover, disulfiram appeared to induce apoptotic cell death in CFPAC-1 cells, increasing the ratio of 7AAD-negative and annexin V-positive cells that represent the populations undergoing early apoptosis (Figure 3C and Figure S3). ALDH1A1 mRNA and protein levels remained persistently decreased in the repopulated cells, indicating that ALDH expression was not restored (Figure 3D-E). Collectively, these results highlight the potential of disulfiram to selectively and efficiently target and eliminate CSCs that would otherwise be resistant to standard anticancer treatments. Furthermore, we performed *in vitro* colony forming assay using disulfiram-pretreated CFPAC-1 cells. As expected, disulfiram-pretreated cells formed colonies much less frequently than control cells did (Figure S3). 

### Oral administration of disulfiram selectively removes ALDH-high cancer cells and inhibits tumor growth in combination with low-dose gemcitabine

**Figure 4:**
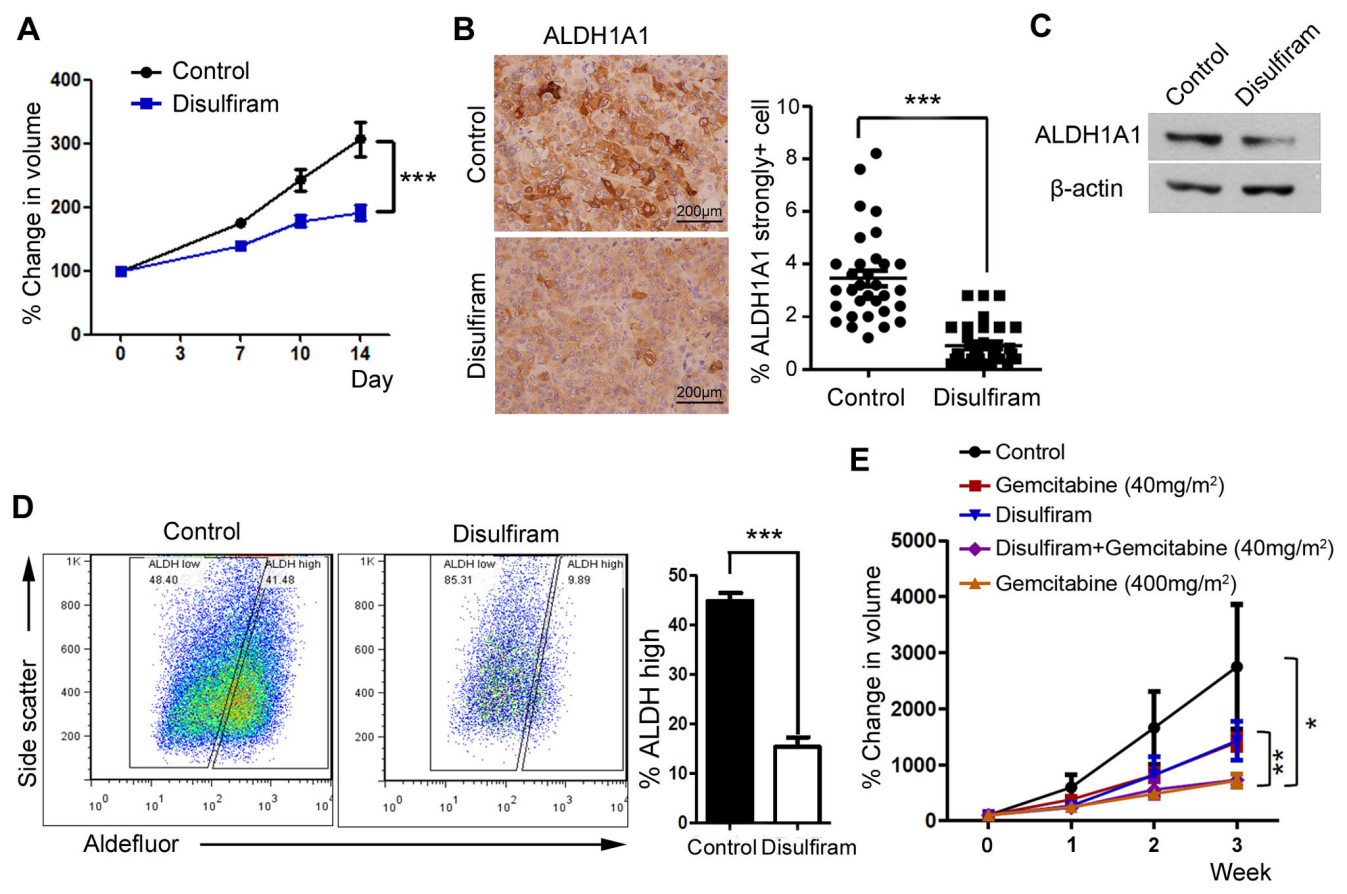
Comparison of *in* *vivo* therapeutic responses after treatment of mice with disulfiram and/or gemcitabine. (A) The growth rate of CFPAC-1 xenografts was measured and presented as percentage change in volume after treating with disulfiram (300 mg/m^2^, P.O, n=4). (B) Histologic analysis and quantification comparing the frequency of ALDH1A1 strongly positive cells in control and disulfiram-treated CFPAC-1 xenografted mice. Graph shows quantification of ALDH1A1 strongly positive cells under X400 magnification in control and disulfiram-treated groups. (C) Representative Western blot showing ALDH1A1 protein expression in control and disulfiram-treated tumors. (D) Aldefluor assay of ALDH activity in dissociated cancer cells retrieved from control and disulfiram-treated CFPAC-1 xenografts. (E) Growth rate of CFPAC-1 xenografts was measured and presented as the percentage change in volume after treatment of mice with vehicle (control), disulfiram (300 mg/m^2^, P.O), low-dose gemcitabine (40 mg/m^2^, I.P), high-dose gemcitabine (400 mg/m^2^, I.P), or disulfiramand low-dose gemcitabine together (n ≥ 3). *P < 0.05, **P < 0.005, and ***P < 0.0005.

To test the effect of disulfiram *in vivo*, we subcutaneously injected mice with CFPAC-1 cancer cells and divided the xenotransplanted mice into disulfiram-treated and corn oil-treated control groups. Tumor growth was significantly delayed by the administration of disulfiram (Figure 4A). IHC for ALDH1A1 of xenograft tumors revealed that the frequency of ALDH1A1 strongly positive cancer cells was markedly reduced by disulfiram-treatment (Figure 4B). We counted the cancer cells in the 10 high-power fields (X400) of the representative slide from xenograft tumors of control (n=3) and disulfiram treated (n=3) groups. ALDH1A1 protein levels were also decreased in disulfiram-treated tumors (Figure 4C). A flow cytometric analysis of the ALDH-expressing cells dissociated from tumors revealed a shift to left of the distribution in disulfiram-treated mice, indicating that ALDH activity was repressed by disulfiram (Figure 4D and Figure S4). We performed *in vivo* tumorigenicity assays using disulfiram-pretreated CFPAC-1 cells. As expected, disulfiram-pretreated cells had a tendency to generate lesser nodules when compared with corresponding control cells (Figure S4, P<0.05 ).

Finally, we explored for the potential benefits of combined disulfiram and gemcitabine therapy. Several previous clinical trials have adopted combination therapies based on low-dose gemcitabine protocols to avoid severe toxicities [29,39-41]. Similarly, we designed a protocol that adopted low-dose gemcitabine [29] along with oral disulfiram. Encouragingly, the combination of low-dose gemcitabine and disulfiram efficiently suppressed tumor growth to a degree comparable to that of a 10-times higher dose of gemcitabine (Figure 4E). Therefore, we conclude that disulfiram not only could achieve additive/synergistic anticancer effects during combination therapy in our *in vivo* PDAC models, but would also enable a significant reduction in the required dose of cytotoxic agents.

## Discussion

The treatment of PDAC is extremely challenging, as this tumor is frequently refractory to conventional anticancer therapies. Most patients present with an unresectable tumor when initially diagnosed; therefore, intensive neo-adjuvant and/or post-operative chemotherapy/CCRT is indicated for the majority of patients on a palliative purpose. In the current study, we described a scenario by which PDAC becomes intractable to anticancer treatments from the perspective of CSCs. In combination with previous studies [12-18,37], our *in vitro*, *in vivo*, and human study results indicate that PDAC CSCs express significantly elevated levels of ALDH and resistance to conventional chemotherapy. 

The reason why ALDH expression is enhanced in pancreatic stem/progenitor cells and PDAC CSCs remains unclear. ALDH is highly expressed in primitive cells during embryogenesis and in stem/progenitor cells in several organs including the pancreas after birth [36]. In a similar context, we speculated that PDAC CSCs, being a primitive form of cancer cells, might express increased levels of ALDH protein. Multiple studies have demonstrated that hematopoietic stem cells and cancer cells of certain solid organs with increased expression of ALDH protein are resistant to alkylating agents and xenobiotics, respectively [42-47]. In this study, we newly found that ALDH strongly positive cancer cells were more abundant in tumors from patients who had previously received chemotherapy/CCRT compared with those who did not receive preoperative treatment. We believe that this observation supports our hypothesis, that therapy-resistant, ALDH strongly positive cancer cells can become enriched by anticancer therapy during actual practice. 

In the current study, we demonstrated that disulfiram exerts a significant cytotoxic effect on ALDH-high cancer cells derived from human PDAC cell lines. However, xenograft experiments showed that disulfiram only slowed the rate of tumor growth, and failed to achieve an actual reduction in the size of tumors. We believe that further optimization of the protocol adopting increased disulfiram and/or gemcitabine concentrations (but still at lower levels than that of the full dose) could be attempted to derive more potent anti-cancer effects. Although disulfiram effectively suppressed the ALDH-high population of cancer cells, the ALDH-low portion was not affected and continued to grow. Such observations led us to conclude that disulfiram could be used in conjunction with other conventional chemotherapy agents to establish a combination protocol designed to exploit the complementary actions of both: disulfiram could suppress the ALDH-high, therapy-resistant CSC population, while conventional anticancer agents could target the disulfiram-insensitive, ALDH-low tumor cells in an additive and/or synergistic manner.

This is also meaningful in that disulfiram could significantly relieve the burden of standard chemo-radiation therapies by reducing the required dosage of toxic chemotherapeutic agents and harmful radiation. Blackstock et al. proposed that a schedule of twice-weekly gemcitabine at only 4% of a full dosage (40 mg/m^2^) retained significant activity when administered concurrently with radiation in patients with advanced PDAC [29]. Using this reduced dosage of gemcitabine in combination with disulfiram, we demonstrated a significant suppression of tumor growth. Myelosuppression is a major dose-limiting toxicity of gemcitabine and it is expected to occur more frequently following the use of gemcitabine with classical cytostatics [4]. To assess the presence of any potential effect on the bone marrow cells, we performed *in vivo* experiments with disulfiram and/or low-dose gemcitabine. As presented in Figure S5, the administration of disulfiram alone or in combination with low dose gemcitabine did not induce significant alterations on the hematopoietic stem cells.

These latter observations are particularly important because disulfiram within therapeutic dose range is a non-cytotoxic agent that does not fall into any category of currently used anticancer agents, and therefore might possibly represent a new method of anti-CSC chemotherapy. Although carbon disulfide, a disulfiram metabolite, is responsible for the behavioral and neurological side effects of disulfiram, most of these presented neurological and psychiatric problems in the case of disulfiram overdose [48]. Richard K. Fuller and Enoch Gordis reviewed the literature on disulfiram, focusing on clinical safety and efficacy studies of this drug alone or in combination with other pharmacotherapeutics [49].

Therefore, we anticipate that disulfiram will be established as a safe and effective agent for use in multi-agent and/or dose-reduced chemotherapy regimens, even in severely compromised patients. The fact that disulfiram was effective when administrated orally is also encouraging, since this feature allows physicians to design a more convenient and flexible protocol.

In conclusion, we demonstrated that PDAC tumor cells with enhanced expression of ALDH possess CSC features. This characteristic not only provides a theoretical explanation for the frequent development of resistance to anticancer treatments, but also forms the basis of a clinically relevant therapeutic strategy insofar as these ALDH-high cancer cells might be effectively suppressed by disulfiram. In this latter context, disulfiramin combination with a conventional anticancer agent provided a safe and effective anticancer therapy *in vivo* in PDAC tumor models.

## Supporting Information

Table S1 **Clinical assessment and ALDH1A1 expression profiles of 57 human PDAC cases.**
(DOC)

Figure S1 **Immunohistochemical analysis of ALDH1A1 in surgical specimens from human PDAC.** Representative photograph demonstrating ALDH1A1 negative (blue dotted line), weakly positive (red dotted line), and strongly positive (red arrows) cancer cells compared with cells used as a positive internal control (blue arrows). Original magnification, X200.
(TIF)

Figure S2 **Distinct subsets of cancer cells can be identified in PDAC-derived cell lines based on ALDH levels.** A. ALDH activity levels in various cell lines derived from human pancreas measured by flow cytometric analyses. B. Representative Western blot analysis for ALDH1A1 protein in four human PDAC cell lines. C. ALDH1A1 mRNA expression measured by semi-quantitative and quantitative RT-PCAR, respectively, in CFPAC-1. D. Western blot demonstrating ALDH1A1 protein levels in CFPAC-1 cells. E. Immunohistochemical staining for ALDH1A1 in CFPAC-1 xenograft tumors. ALDH^Br^, ALDH bright, and ALDH^Ng^, ALDH negative. *P < 0.05.
(TIF)

Figure S3 **PDAC-derived cell lines with higher ALDH activity are more sensitive to disulfiram.** A. Viability assay in PDAC-derived cells (left panel) and normal pancreatic ductal cell (right panel) treated with disulfiram. B. Flow cytometric assay for 7AAD-AnnexinV+ early apoptotic cells after 12-hour treatment with disulfiram (10μM). C. Graphs comparing colony forming ability of disulfiram-pretreated CFPAC-1 cells. **P<0.005.
(TIF)

Figure S4 A. Table comparing Aldefluor assay results demonstrating changes in cancer cell distribution of disulfiram -treated tumors. DSF, disulfiram. B. Graphs comparing *in* *vivo* tumorigenicity of disulfiram-pretreated CFPAC-1 cells.
(TIF)

Figure S5 **Comparison of murine LSK population after DSF and/or low-dose GCB administration.** A. Comparison of murine LSK population after DSF (7mg/kg, twice weekly, I.P) administration. The x-axis indicates post-injection time. B. Comparing LSK frequency of DSF and/or low-dose GCB (40mg/m^2^, weekly, I.P) treated mouse after 2 weeks. LSK, lineage- Sca+ c-kit+. BM, bone marrow. DSF, disulfiram. GCB, gemcitabine.
(TIF)
