# Supplementary material for: Reversing the Intractable Nature of Pancreatic Cancer by Selectively Targeting ALDH-High, Therapy-Resistant Cancer Cells
Source: PLoS One. 2013 Oct 23;8(10):e78130. doi: 10.1371/journal.pone.0078130 (PMC3806801; doi:10.1371/journal.pone.0078130)
Supplement: Table S1 — Clinical assessment and ALDH1A1 expression profiles of 57 human PDAC cases. (DOC) [file pone.0078130.s001.doc]

**Table S1**

| Case  No. | Preoperative therapy | | Stage | | | | Postop. CCRT* | ALDH1A1 | | |
| --- | --- | --- | --- | --- | --- | --- | --- | --- | --- | --- |
|  |  |  | Group | TNM | | |  | Strongly  Positive | Weakly  Positive | Negative |
|  | Radiation | Chemotherapy |  |  |  |  |  |  |  |  |
|  |  |  |  | T | N | M |  |  |  |  |
| 1 | No | Gemzar† | IIB | 3 | 1 | 0 | No | X | O | O |
| 2 | Yes | Gemzar | IIA | 3 | 0 | 0 | No | O | O | X |
| 3 | Yes | Gemzar | IIA | 3 | 0 | 0 | Yes | O | X | O |
| 4 | Yes | Gemzar+Cisplan | IIA | 3 | 0 | 0 | Yes | O | O | O |
| 5 | No | 5-FU+DDP‡ | IIA | 3 | 0 | 0 | Yes | O | X | X |
| 6 | No | Gemzar+DDP | IIA | 3 | 0 | 0 | No | O | X | O |
| 7 | No | Gemzar+DDP | IIB | 3 | 1 | 0 | Yes | O | X | O |
| 8 | No | Gemzar+DDP | IIA | 3 | 0 | 0 | Yes | O | O | X |
| 9 | No | Gemzar | IIA | 3 | 0 | 0 | No | X | X | O |
| 10 | No | Gemzar+DDP | IIA | 3 | 0 | 0 | Yes | X | X | O |
| 11 | No | Gemzar | IIA | 3 | 0 | 0 | No | O | O | O |
| 12 | No | Gemzar+DDP | IIA | 3 | 0 | 0 | Yes | O | O | X |
| 13 | Yes | Gemzar+DDP | IIB | 3 | 1 | 0 | No | O | O | X |
| 14 | Yes | Gemzar+DDP | IIA | 3 | 0 | 0 | Yes | O | O | X |
| 15 | Yes | Ts-1§ | IIB | 3 | 1 | 0 | No | X | O | O |
| 16 | No | Gemzar+Ts-1 | IIB | 3 | 1 | 0 | Yes | O | O | X |
| 17 | Yes | Gemzar | IIB | 3 | 1 | 0 | No | O | O | X |
| 18 | Yes | Gemzar | IIB | 3 | 1 | 0 | No | O | O | O |
| 19 | Yes | Gemzar+DDP | IIA | 3 | 0 | 0 | Yes | O | O | X |
| 20 | No | Gemzar | IIA | 3 | 0 | 0 | Yes | O | O | O |
| 21 | No | No | IIA | 3 | 0 | 0 | Yes | O | O | X |
| 22 | No | No | IIA | 3 | 0 | 0 | Yes | X | O | O |
| 23 | No | No | IIB | 3 | 1 | 0 | No | O | X | X |
| 24 | No | No | IIA | 3 | 0 | 0 | Yes | X | O | O |
| 25 | No | No | IIB | 3 | 1 | 0 | Yes | O | X | O |
| 26 | No | No | IIB | 3 | 1 | 0 | Yes | O | X | X |
| 27 | No | No | IIB | 3 | 1 | 0 | Yes | O | X | O |
| 28 | No | No | IIB | 3 | 1 | 0 | Yes | X | O | O |
| 29 | No | No | IIA | 3 | 0 | 0 | No | O | X | O |
| 30 | No | No | IIB | 3 | 1 | 0 | Yes | O | O | X |

| Case  No. | Preoperative therapy | | Stage | | | | Postop. CCRT | ALDH1A1 | | |
| --- | --- | --- | --- | --- | --- | --- | --- | --- | --- | --- |
|  |  |  | Group | TNM | | |  | Strongly  Positive | Weakly  Positive | Negative |
|  | Radiation | Chemotherapy |  |  |  |  |  |  |  |  |
| 31 | No | No | IIB | 3 | 1 | 0 | No | O | O | X |
| 32 | No | No | IIB | 3 | 1 | 0 | Yes | O | X | O |
| 33 | No | No | IIA | 3 | 0 | 0 | No | X | X | O |
| 34 | No | No | IIB | 3 | 1 | 0 | Yes | X | X | O |
| 35 | No | No | IIB | 3 | 1 | 0 | No | X | X | O |
| 36 | No | No | IIB | 3 | 1 | 0 | Yes | X | O | O |
| 37 | No | No | IIA | 3 | 0 | 0 | No | O | O | O |
| 38 | No | No | IIB | 2 | 1 | 0 | No | O | X | O |
| 39 | No | No | IIB | 3 | 1 | 0 | Yes | X | X | O |
| 40 | No | No | IIA | 3 | 0 | 0 | Yes | O | O | X |
| 41 | No | No | IIA | 3 | 0 | 0 | No | X | O | O |
| 42 | No | No | IIB | 3 | 1 | 0 | Yes | X | O | O |
| 43 | No | No | IIA | 3 | 0 | 0 | Yes | X | X | O |
| 44 | No | No | IIB | 3 | 1 | 0 | No | X | X | O |
| 45 | No | No | IIA | 3 | 0 | 0 | No | X | X | O |
| 46 | No | No | IIB | 3 | 1 | 0 | No | O | X | O |
| 47 | No | No | IIB | 3 | 1 | 0 | Yes | X | X | O |
| 48 | No | No | IIB | 3 | 1 | 0 | Yes | X | O | X |
| 49 | No | No | IIB | 3 | 1 | 0 | Yes | O | O | X |
| 50 | No | No | IIB | 3 | 1 | 0 | Yes | O | O | X |
| 51 | No | No | IIA | 3 | 0 | 0 | Yes | X | O | X |
| 52 | No | No | IIA | 3 | 0 | 0 | No | X | O | O |
| 53 | No | No | IIB | 3 | 1 | 0 | Yes | X | O | X |
| 54 | No | No | IIB | 3 | 1 | 0 | Yes | O | O | X |
| 55 | No | No | IIB | 3 | 1 | 0 | Yes | O | O | X |
| 56 | No | No | IIA | 3 | 0 | 0 | Yes | O | O | X |
| 57 | No | No | IIA | 3 | 0 | 0 | Yes | O | O | X |

*Postop. CCRT: postoperative chemo-radiation therapy.

†Gemzar: gemcitabine.

‡DDP: cisplan.

§TS-1: combination of tegafur, gimeracil and oteracil potassium.
