# Supplementary figures and images for: Reversing the Intractable Nature of Pancreatic Cancer by Selectively Targeting ALDH-High, Therapy-Resistant Cancer Cells
Source: PLoS One. 2013 Oct 23;8(10):e78130. doi: 10.1371/journal.pone.0078130 (PMC3806801; doi:10.1371/journal.pone.0078130)

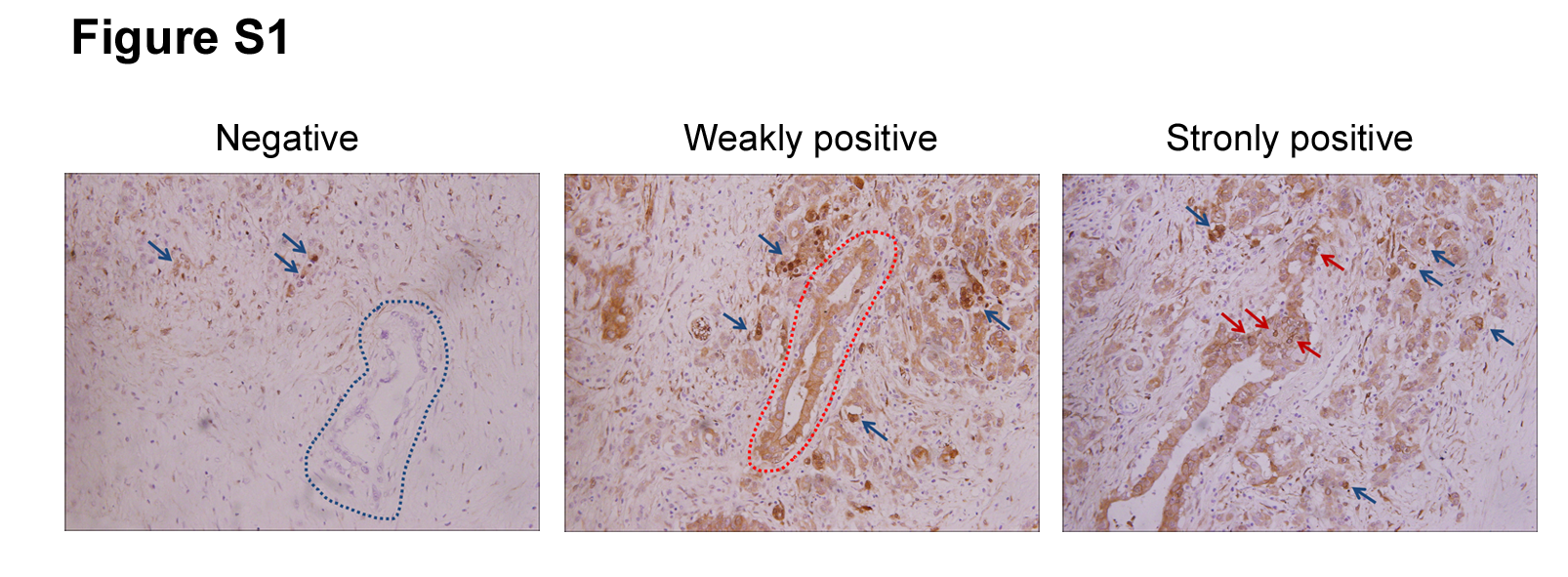

Supplement: Figure S1 — Immunohistochemical analysis of ALDH1A1 in surgical specimens from human PDAC. Representative photograph demonstrating ALDH1A1 negative (blue dotted line), weakly positive (red dotted line), and strongly positive (red arrows) cancer cells compared with cells used as a positive internal control (blue arrows). Original magnification, X200. (TIF) [file pone.0078130.s002.tif]

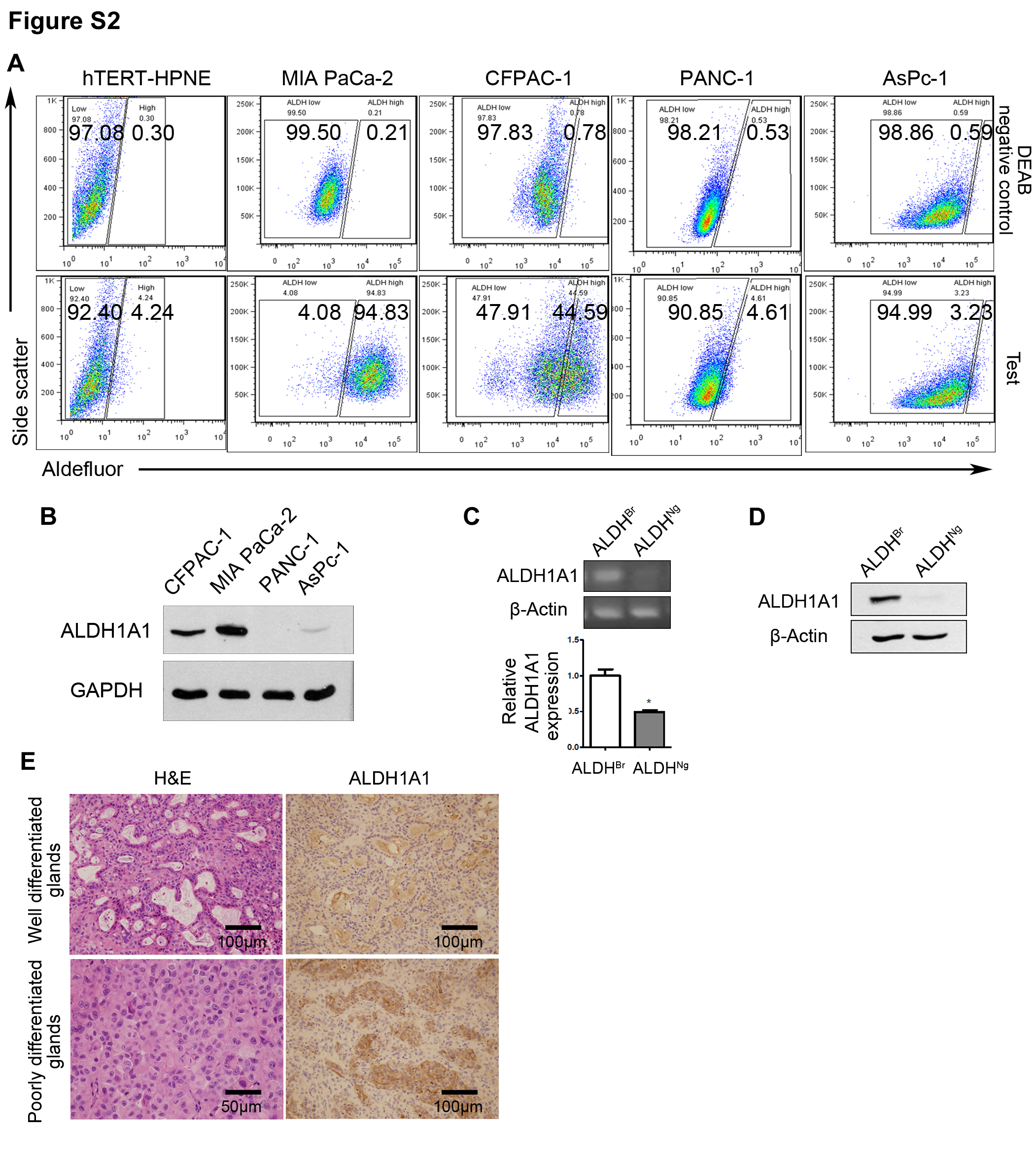

Supplement: Figure S2 — Distinct subsets of cancer cells can be identified in PDAC-derived cell lines based on ALDH levels. A. ALDH activity levels in various cell lines derived from human pancreas measured by flow cytometric analyses. B. Representative Western blot analysis for ALDH1A1 protein in four human PDAC cell lines. C. ALDH1A1 mRNA expression measured by semi-quantitative and quantitative RT-PCAR, respectively, in CFPAC-1. D. Western blot demonstrating ALDH1A1 protein levels in CFPAC-1 cells. E. Immunohistochemical staining for ALDH1A1 in CFPAC-1 xenograft tumors. ALDHBr, ALDH bright, and ALDHNg, ALDH negative. *P < 0.05. (TIF) [file pone.0078130.s003.tif]

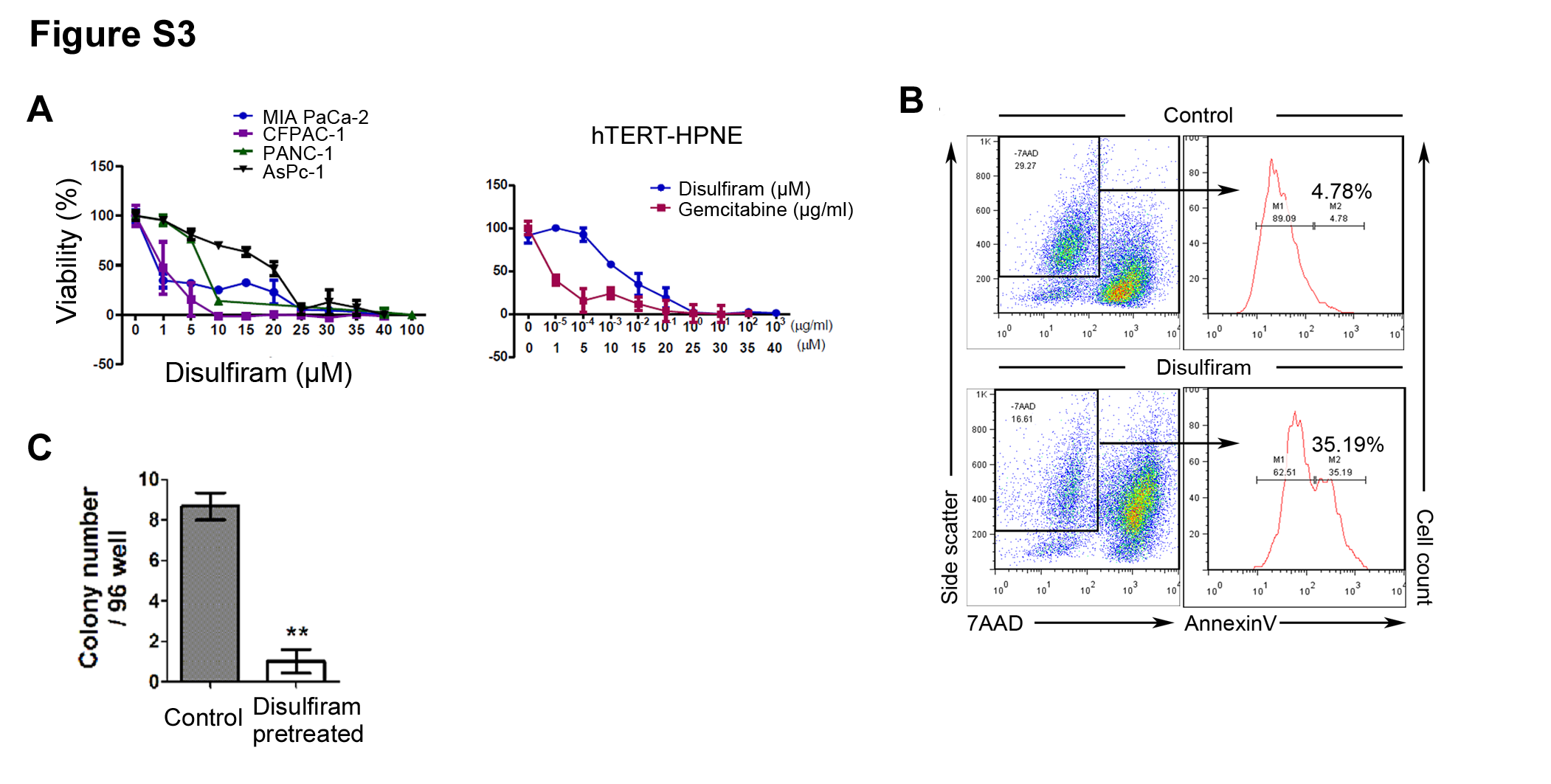

Supplement: Figure S3 — PDAC-derived cell lines with higher ALDH activity are more sensitive to disulfiram. A. Viability assay in PDAC-derived cells (left panel) and normal pancreatic ductal cell (right panel) treated with disulfiram. B. Flow cytometric assay for 7AAD-AnnexinV+ early apoptotic cells after 12-hour treatment with disulfiram (10μM). C. Graphs comparing colony forming ability of disulfiram-pretreated CFPAC-1 cells. **P<0.005. (TIF) [file pone.0078130.s004.tif]

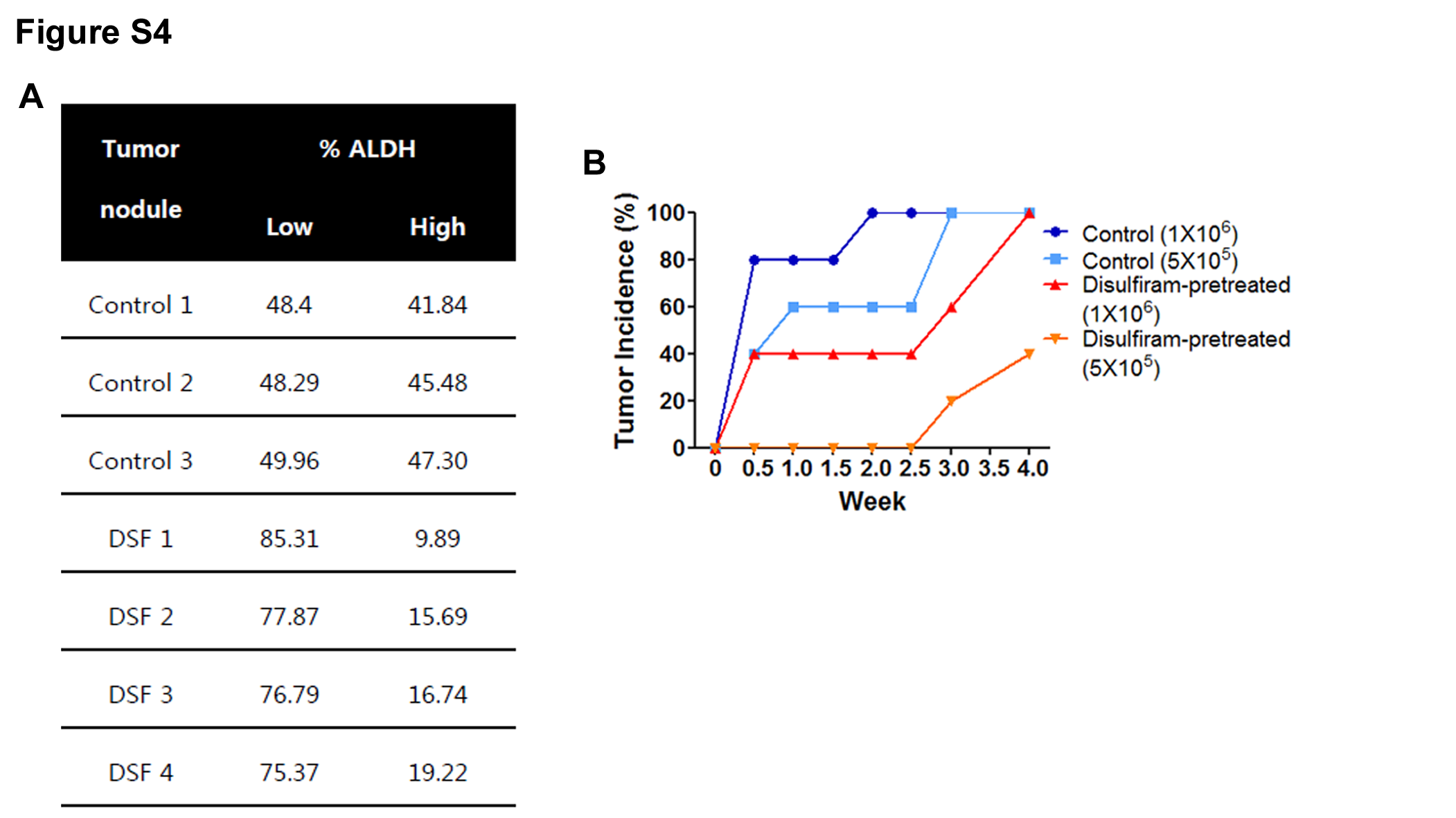

Supplement: Figure S4 — A. Table comparing Aldefluor assay results demonstrating changes in cancer cell distribution of disulfiram -treated tumors. DSF, disulfiram. B. Graphs comparing in vivo tumorigenicity of disulfiram-pretreated CFPAC-1 cells. (TIF) [file pone.0078130.s005.tif]

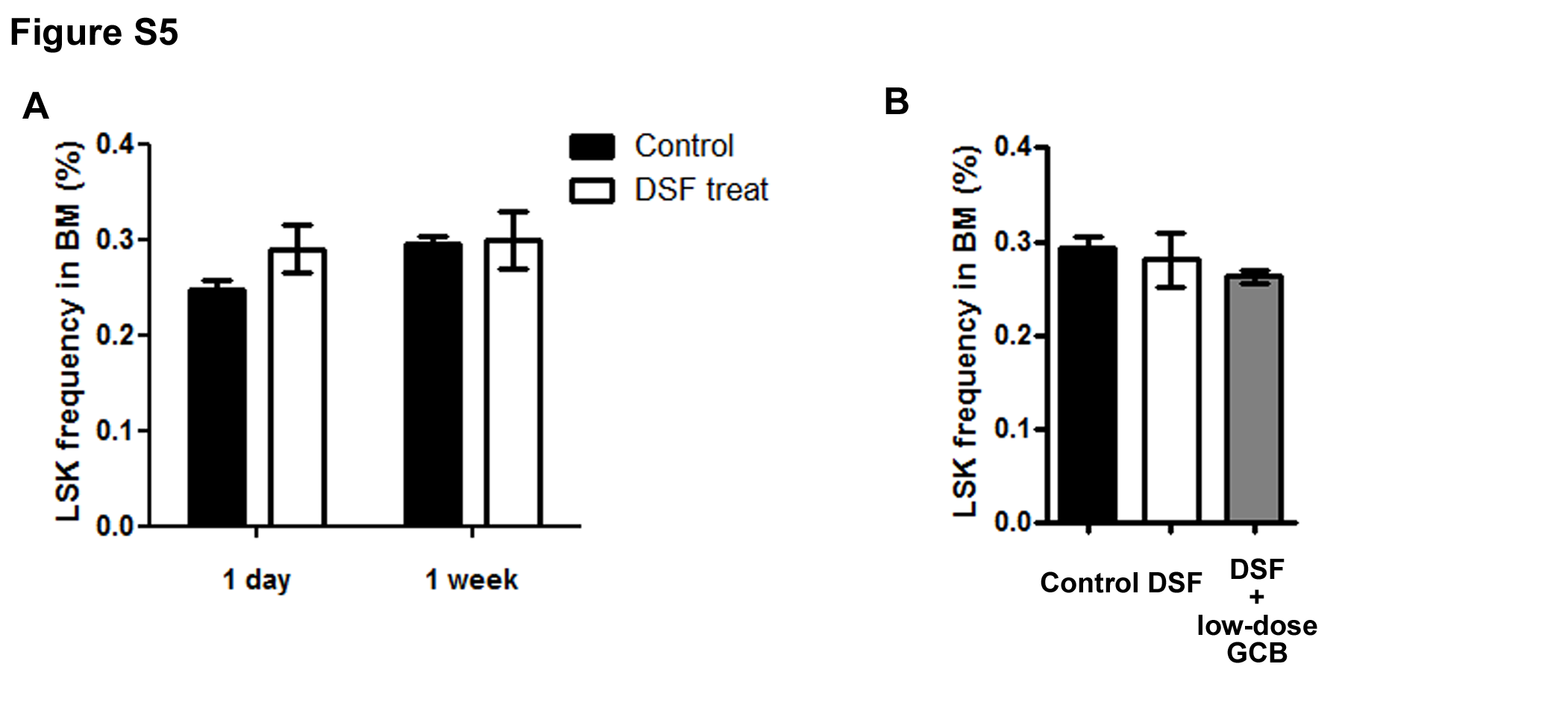

Supplement: Figure S5 — Comparison of murine LSK population after DSF and/or low-dose GCB administration. A. Comparison of murine LSK population after DSF (7mg/kg, twice weekly, I.P) administration. The x-axis indicates post-injection time. B. Comparing LSK frequency of DSF and/or low-dose GCB (40mg/m2, weekly, I.P) treated mouse after 2 weeks. LSK, lineage- Sca+ c-kit+. BM, bone marrow. DSF, disulfiram. GCB, gemcitabine. (TIF) [file pone.0078130.s006.tif]
